# Integrated Implementation Strategies to Promote the Use of AI-Assisted Diagnostic Software for Lung Nodule Screening in China: Process Evaluation Based on the RE-AIM Framework

**DOI:** 10.2196/76002

**Published:** 2026-03-24

**Authors:** Xiwen Liao, Yifan Tian, Yaning Cheng, Xiaomeng Sun, Yingxin Zhang, Yan Tang, Zhe Zhao, Yuanyuan Lun, Shentang Wang, Yan Li, Yingzhe Fu, Danrui Zong, Ling Chen, Qimin Wang, Hongxia Zhang, Chen Yao, Di Chen

**Affiliations:** 1 Department of Medical Statistics, Peking University First Hospital, Beijing, China; 2 Clinical Research Institute, Institute of Advanced Clinical Medicine, Peking University, Beijing, China; 3 Rehabilitation Information Research Department, China Rehabilitation Science Institute, No. 18 Jiaomen North Road, Beijing, 100068, China, 86 010-67563322; 4 Tieying Hospital, Fengtai Rehabilitation Hospital of Beijing Municipality, Beijing, China; 5 Beijing Boai Hospital, China Rehabilitation Research Center, Beijing, China

**Keywords:** diagnostic support, artificial intelligence, implementation strategies, RE-AIM, hierarchical medical system

## Abstract

**Background:**

While artificial intelligence (AI)–assisted diagnostic software holds promise for improving diagnostic efficiency and reducing disparities in health care delivery, its effective implementation in lower-tier health care settings remains limited in China. Most existing studies have focused on algorithm performance, while real-world implementation strategies remain underexplored, particularly in resource-constrained clinical environments.

**Objective:**

This study aimed to design, implement, and evaluate an integrated, context-specific strategy to facilitate the effective implementation of AI-assisted diagnostic software for pulmonary nodule screening in a secondary hospital within China’s hierarchical health care system.

**Methods:**

A prospective process evaluation was conducted in a secondary hospital in Beijing, supported by a collaborating tertiary referral center. The implementation strategy integrated AI software for computed tomography–based pulmonary nodule analysis into the diagnostic workflow of the secondary hospital, enabling initial screening and identification of suspected cases. Patients meeting referral criteria were referred to the tertiary hospital through a structured mechanism facilitated by a cloud-based data transfer tool, which enabled the return of diagnostic feedback and ensured continuity through a bidirectional referral and feedback system. Short-term implementation outcomes were evaluated using the RE-AIM framework, focusing on feasibility, adoption, and areas for improvement.

**Results:**

During the study period, 85.6% (1105/1291) of chest computed tomography scans were analyzed using the AI software, with a significant increase in the pulmonary nodule detection rate compared to the historical control group (65.2% vs 32.4%, *P*<.001). Among eligible patients, 88% (22/25) completed referral to the tertiary hospital, indicating a high level of adherence to the referral protocol. Moreover, 90.9% (20/22) of imaging data were transmitted successfully via the data transfer tool, facilitating timely diagnosis. However, several challenges remained, including the low rate of fully documented referral records (28%) and minimal use of diagnostic feedback by referring physicians. These limitations were largely attributed to disruptions in routine clinical workflows due to inadequate integration of the data transfer tool with existing hospital systems and continued reliance on manual data entry.

**Conclusions:**

This study demonstrated the feasibility and potential value of deploying AI-assisted diagnostic software in a secondary hospital when supported by a tailored referral mechanism and interhospital data exchange systems. The findings highlighted the critical role of referral adherence, information infrastructure, and feedback mechanisms in optimizing the clinical utility of AI technologies. Further multicenter research is warranted to assess the generalizability, cost-effectiveness, long-term sustainability, and scalability of the implementation strategies across diverse health care settings.

## Introduction

China implemented a hierarchical medical system (HMS) in 2015 to optimize health care resource allocation and address the disproportionate distribution of patient volume across the multitiered health care infrastructure [1-3]. The system is structured to align the severity of a patient’s condition with the appropriate level of care, thereby promoting both efficiency and equity. Under this framework, primary care facilities, such as community health centers in urban areas and village clinics in rural areas, serve as the first point of contact, providing basic health services, preventive care, and management of common illnesses. Secondary hospitals are responsible for managing moderately complex cases and referring critically ill or highly specialized patients to tertiary hospitals, which are equipped to handle severe and advanced medical conditions. Ideally, this structure ensures that each tier functions within its designated scope, facilitating a coordinated and balanced health care delivery system.

In practice, however, the implementation of the HMS has encountered major challenges, particularly at the primary and secondary care levels. Despite ongoing investments in infrastructure and service capacity, these lower-tier institutions continue to be underutilized [4-6]. Contributing factors include limited medical resources, lack of skilled health care personnel, and public perception that care at lower-tier facilities is of suboptimal quality [6-9]. As a result, many patients bypass community- and county-level institutions and seek care directly at tertiary hospitals, assuming that these institutions offer faster and more reliable medical services [2,10,11]. Bypassing lower-tier facilities has disrupted the intended patient flow within the HMS, placing an excessive burden on tertiary hospitals. These institutions face substantial overcrowding because they are required to manage highly specialized cases alongside a large volume of patients with conditions that could be appropriately addressed at lower levels of care. Consequently, strengthening the quality and capacity of care at primary and secondary health care institutions has become a strategic priority for optimizing China’s health care delivery system [11-13].

Artificial intelligence (AI), particularly AI-assisted diagnostic software, provides a promising solution to the challenges faced by China’s HMS. Functioning as a second reader, AI delivers consistent and rapid data interpretation, reducing diagnostic errors, and minimizes variability in care quality [14-16]. However, clinical implementation of AI-assisted diagnostic software in China remains predominantly concentrated in tertiary hospitals [17]. This implementation pattern is largely driven by institutional capacity, including the availability of information technology infrastructure and financial resources. As a result, adoption has been primarily limited to large, well-resourced tertiary hospitals [17]. Studies indicate that AI-assisted diagnostic software has become a valuable tool in tertiary hospitals for enhancing the diagnostic and clinical decision-making capabilities of junior physicians [18-21]. It has been widely applied across multiple clinical specialties, including pulmonary diseases [19,22,23], thyroid disorders [24,25], ophthalmology [20,21], gastrointestinal diseases [26,27], and orthopedics [28-30]. Furthermore, in high-volume tertiary care settings, AI-assisted diagnostic software has demonstrated the ability to reduce diagnostic time and improve overall workflow efficiency [22,31,32].

However, the clinical value of AI-assisted diagnostic software for senior physicians remains controversial. While some studies suggest that in complex cases, particularly those involving ambiguous or difficult-to-interpret imaging features, the use of AI can enhance diagnostic accuracy among experienced clinicians [31,33,34], others have found no significant improvement in diagnostic accuracy among senior physicians using AI-assisted diagnostic software [24,35-37]. These findings underscore the limitations of the tertiary hospital–centered implementation mode, which may not fully leverage the potential clinical value of AI technologies, especially in more diverse and complex real-world settings. More importantly, the current implementation model also hinders broader application of AI-assisted diagnostic software in primary and secondary health care institutions, where such tools may be particularly valuable in addressing resource constraints and improving diagnostic capabilities.

To advance effective implementation, a previous study used the updated Consolidated Framework for Implementation Research (CFIR) to qualitatively identify key barriers to implementing AI-assisted diagnostic software in China [38]. Building upon these findings, this study aimed to develop context-specific implementation strategies for AI-assisted diagnostic software, with pulmonary nodule screening serving as a representative clinical scenario. The strategies focused on aligning AI deployment with the clinical needs of lower-tier hospitals, establishing coordinated referral pathways to tertiary hospitals, enabling standardized and secure interhospital data sharing, and implementing diagnostic feedback mechanisms to support continuity of care. The real-world feasibility and preliminary effectiveness of these strategies were evaluated to identify areas for further implementation refinement.

## Methods

### Development of Implementation Strategies

#### Overview

The integrated implementation strategies were developed based on key barriers identified through a prior CFIR-guided qualitative interview study [38], as summarized in Table 1. These barriers were identified and prioritized using a 3-round modified Delphi process. In the first round, qualitative interviews were conducted to comprehensively elicit potential barriers, followed by 2 rounds of structured ranking to select the top 5 barriers most relevant to real-world implementation.

The Expert Recommendations for Implementing Change (ERIC) compilation was employed to develop implementation strategies tailored to the identified barriers [39,40]. As a foundational resource in implementation science, ERIC provides an extensive compilation of 73 implementation strategies across multiple domains. These strategies were developed through a Delphi consensus process involving the systematic engagement of stakeholders from both clinical practice and implementation research. The ERIC compilation serves as a robust theoretical basis for researchers and practitioners to select and apply context-specific strategies for implementing innovations, including AI-assisted diagnostic software.

**Table 1.** Main barriers to implementing AI-assisted diagnostic software in China.

Main barriers	Short description
Unsatisfactory clinical performance at tertiary hospitals	The AI^a^-assisted diagnostic software was predominantly implemented in tertiary hospitals, but its clinical performance was suboptimal when handling complex cases.
Misalignment between software functions and goals of tertiary hospitals	Existing AI-assisted diagnostic software functions, focused on population-level screening, did not align with tertiary hospitals’ goals of providing specialized care for complex conditions.
Lack of a collaborative network between primary/secondary and tertiary hospitals	A referral mechanism for patients with positive or indeterminate results from primary or secondary hospitals to tertiary hospitals was not fully established.
Lack of information security measures and certification	The AI-assisted diagnostic software and interhospital data transfer lacked the required information protection qualifications and confidentiality measures.
Lack of performance feedback and evaluation	Insufficient evaluation of the AI-assisted diagnostic software’s real-world diagnostic accuracy hindered optimization efforts and undermined user trust.

a AI: artificial intelligence.

In this study, implementation strategies were initially derived from the ERIC compilation and further refined using the CFIR-ERIC mapping tool to ensure both scientific validity and practical feasibility [41]. This publicly available, evidence-based tool is designed to match specific implementation barriers identified through the CFIR to relevant ERIC strategies [42]. By systematically linking barriers to targeted strategies, this tool supported the initial prioritization of evidence-informed strategies, which were subsequently refined and adapted to specific implementation contexts.

The implementation strategies evaluated in this study were developed using the following multistage process:

#### CFIR-ERIC Mapping and Initial Prioritization

CFIR-ERIC mapping was conducted to systematically link previously identified CFIR-based implementation barriers to candidate ERIC strategies, with endorsement levels quantified using cumulative percentages (Multimedia Appendix 1). Strategies with cumulative endorsement exceeding 50% were identified as high-priority candidates and were selected for further contextual evaluation.

#### Stakeholder Recruitment and Consensus Preparation

Building on these results, stakeholders who had participated in the previous CFIR-guided qualitative interview were invited to participate in a follow-up consensus discussion. Of the 40 participants invited, 27 (67.5%) participated in the consensus discussion, including 16 clinicians, 3 hospital administrators, 4 hospital informatics specialists, and 4 AI vendors, all with direct experience in AI-assisted diagnostic workflows. The consensus session began with a presentation of the key implementation barriers, their corresponding CFIR constructs, and the mapped ERIC strategies identified as high-priority candidates. To ensure a shared understanding and reduce interpretive variability, standardized definitions and practical examples of each ERIC strategy were provided prior to discussion.

#### Consensus Decision-Making and Strategy Selection

Participants then engaged in strategy-by-strategy discussions to appraise each candidate ERIC strategy. Evaluation was guided by predefined criteria, including (1) contextual relevance to lower-tiered hospital settings in China, (2) feasibility of implementation within existing clinical workflows and resource constraints, and (3) the logical and practical capacity of the strategy to address the corresponding CFIR-identified barriers. For each strategy, participants discussed anticipated implementation requirements, potential operational challenges, and alignment with current interinstitutional collaboration processes, data governance structures, and clinician responsibilities.

Disagreements were addressed through iterative, facilitated discussion aimed at clarifying underlying assumptions, contextual constraints, and anticipated implementation pathways. When consensus could not be achieved through discussion alone, a structured decision rule was applied. Participants were asked to indicate whether each strategy should be retained or excluded based on the predefined evaluation criteria. Strategies receiving support from more than half of participating stakeholders were retained for integration into the final implementation package.

#### Illustrative Example of Barrier-to-Strategy Translation

For example, the barrier “lack of information security measures and certification,” categorized under the CFIR domain *External Policies and Incentives*, reflected challenges in obtaining recognized information security certification for AI-assisted tools, which hindered secure data exchange between secondary and tertiary hospitals. CFIR-ERIC mapping identified 7 Tier 2 strategies for this barrier, including involving executive boards (41%), altering incentive or allowance structures (41%), building a coalition (33%), capturing and sharing local knowledge (26%), identifying and preparing champions (22%), informing local opinion leaders (22%), and conducting local consensus discussions (22%).

During consensus discussions, participants emphasized that information security certification and cross-institutional data governance are primarily governed by external regulatory requirements and interorganizational coordination mechanisms rather than by internal financial incentives or locally generated experiential knowledge. Accordingly, strategies such as altering incentive or allowance structures and capturing and sharing local knowledge were deprioritized, as they were considered to have limited feasibility and weak logical alignment with the policy-level nature of the barrier.

The strategy of involving executive boards was discussed but was not retained, as participants considered hospital-level executive leadership unlikely to exert meaningful influence over externally regulated data transfer and certification processes within the current implementation context. In contrast, building a coalition with a qualified data transfer platform was prioritized, as it was perceived to support coordinated governance, regulatory compliance, and shared accountability for secure data exchange across participating hospitals.

The ERIC strategies that reached consensus and their corresponding implementation strategies are summarized in Table 2.

**Table 2.** Selected ERIC strategies and corresponding implementation strategies.

Key barrier	CFIR^a^ domain	Mapping outcome	Selected ERIC^b^ strategy	Specific implementation strategy
Unsatisfactory clinical performance at tertiary hospitals	Relative advantage	Tier 1 strategies: 0 Tier 2 strategies: 8	Conduct local consensus discussions	Shift the implementation focus of AI^c^-assisted diagnostic software to primary or secondary hospitals and achieve consensus among key stakeholders.
Misalignment between software functions and goals of tertiary hospitals	Goals and feedback	Tier 1 strategies: 1 Tier 2 strategies: 6	Develop a formal implementation blueprint	Develop an AI implementation plan aligned with the primary or secondary hospital’s clinical needs and goals, including task assignments and timelines.
Lack of a collaborative network between primary or secondary and tertiary hospitals	Cosmopolitanism	Tier 1 strategies: 3 Tier 2 strategies: 5	Build a coalition	Establish a collaborative network to ensure smooth referral of suspected cases from primary or secondary hospitals to tertiary hospitals following AI-assisted screening.
Lack of information security measures and certification	External policy and incentives	Tier 1 strategies: 0 Tier 2 strategies: 7	Build a coalition	Collaborate with information platform providers to enable data sharing for referred patients, establishing clear protocols to ensure data completeness, timeliness, and confidentiality across hospitals.
Lack of performance feedback and evaluation	Reflecting and evaluating	Tier 1 strategies: 2 Tier 2 strategies: 6	Facilitate relay of clinical data to providers	Establish a feedback mechanism for returning final diagnoses from tertiary to primary or secondary hospitals, enabling care continuity and helping clinicians assess AI performance for sustainable use.

a CFIR: Consolidated Framework for Implementation Research.

b ERIC: Expert Recommendations for Implementing Change.

c AI: artificial intelligence.

### Study Design

This prospective, observational, real-world study employed the RE-AIM evaluation framework to preliminarily evaluate the effectiveness and feasibility of implementation strategies designed to integrate AI-assisted diagnostic software into China’s HMS, following 3 months of implementation. The process evaluation focused on identifying practical challenges, successes, areas for optimization, and the potential to enhance patient outcomes and clinical workflows.

### Ethical Considerations

Centralized ethical approval for this study was obtained from the Ethical Review Board of the China Rehabilitation Research Center (approval number: 2024-032-1). Written informed consent was obtained from all patients involved in the referral process. Patient privacy and confidentiality were strictly protected. All potentially identifying information (including names, initials, and patient identification numbers) was removed unless essential for scientific purposes. No identifiable participant information appears in the manuscript or supplementary materials. Participation was entirely voluntary, and no compensation was provided to participants in this study.

### Study Settings and Tools

#### Study Settings

In selecting appropriate primary or secondary hospitals for AI-assisted diagnostic software deployment, the study first established that eligible sites must possess the necessary hardware infrastructure and technical support environment. Insights from the previous qualitative interviews highlighted that the availability of necessary medical equipment meeting the technical requirements was a fundamental facilitator for the successful implementation of AI-assisted diagnostic software. Given that most community health centers in China lack sufficient imaging capabilities, the study prioritized secondary hospitals with more advanced diagnostic equipment as the primary sites for deployment.

The selection process further considered several factors, including the hospital’s technical capacity, the maturity of its collaborative networks with tertiary hospitals, and the willingness to participate in the study. Based on these criteria, 2 hospitals in Beijing were selected as implementation sites: a secondary rehabilitation hospital (Fengtai Rehabilitation Hospital of Beijing) and a general tertiary hospital (China Rehabilitation Research Center). Key characteristics of the participating hospitals are summarized in Table 3.

**Table 3.** Characteristics of participating hospitals.

Characteristic	Fengtai Rehabilitation Hospital of Beijing	China Rehabilitation Research Center
Location	Beijing	Beijing
Funding source	Public	Public
Tier	Secondary	Tertiary
Hospital type	Specialized	General
Specialties	Neurological, geriatric, and bone and joint rehabilitation services	Specialized respiratory, sports injury, and hearing and speech rehabilitation services
Bed capacity	211	1100
Staff number	434	1700
Annual outpatient volume	300,000	470,000

#### AI-Assisted Diagnostic Software

The study used a commercially available AI-assisted screening system for pulmonary nodules developed by HY Medical Technology Co., Ltd. (Beijing, China). This software received approval from the National Medical Products Administration in 2022 and obtained CE certification in 2020. It automatically identifies and segments potential lesions in chest computed tomography (CT) scans, providing a malignancy score ranging from 0% to 100% to assist physicians in assessing nodule risk. Additional details on the functionalities of the AI-assisted diagnostic software are provided in Multimedia Appendix 2.

The AI-assisted diagnostic software for pulmonary nodules was locally deployed at the secondary hospital and seamlessly integrated with the hospital’s picture archiving and communication system (PACS), which supported CT image analysis and assisted clinical decision-making without disrupting routine diagnostic workflows.

#### Data Transfer Tool

To address fragmented information systems between the participating hospitals, the electronic source data repository (ESDR) system was employed to facilitate seamless patient data transfer. Developed by Hangzhou LionMed Medical Information Technology Co., Ltd, the ESDR system is designed to standardize real-world data collection, governance, and management, enhancing clinical research and health care interoperability. Functions and benefits of the ESDR system have been well-validated in previous studies [43-45].

The ESDR tool operated on a secure, cloud-based platform managed by the tertiary hospital, with encrypted external access links provided to both participating hospitals to ensure secure, real-time data sharing. Using the CT image ID, AI-generated results, including pulmonary nodule risk scores, clinical feature data, and chest CT images, were automatically uploaded from the PACS to the ESDR platform. This enabled physicians at the tertiary hospital to dynamically access and review CT images and diagnostic data directly on their terminal devices. Drawing on this information and their clinical judgment, physicians determined the final diagnosis and developed follow-up plans. The diagnostic decisions and follow-up strategies were recorded in the ESDR tool, supporting dynamic follow-up at 1, 3, 6, and 12 months.

### Implementation Process

**Figure 1.**
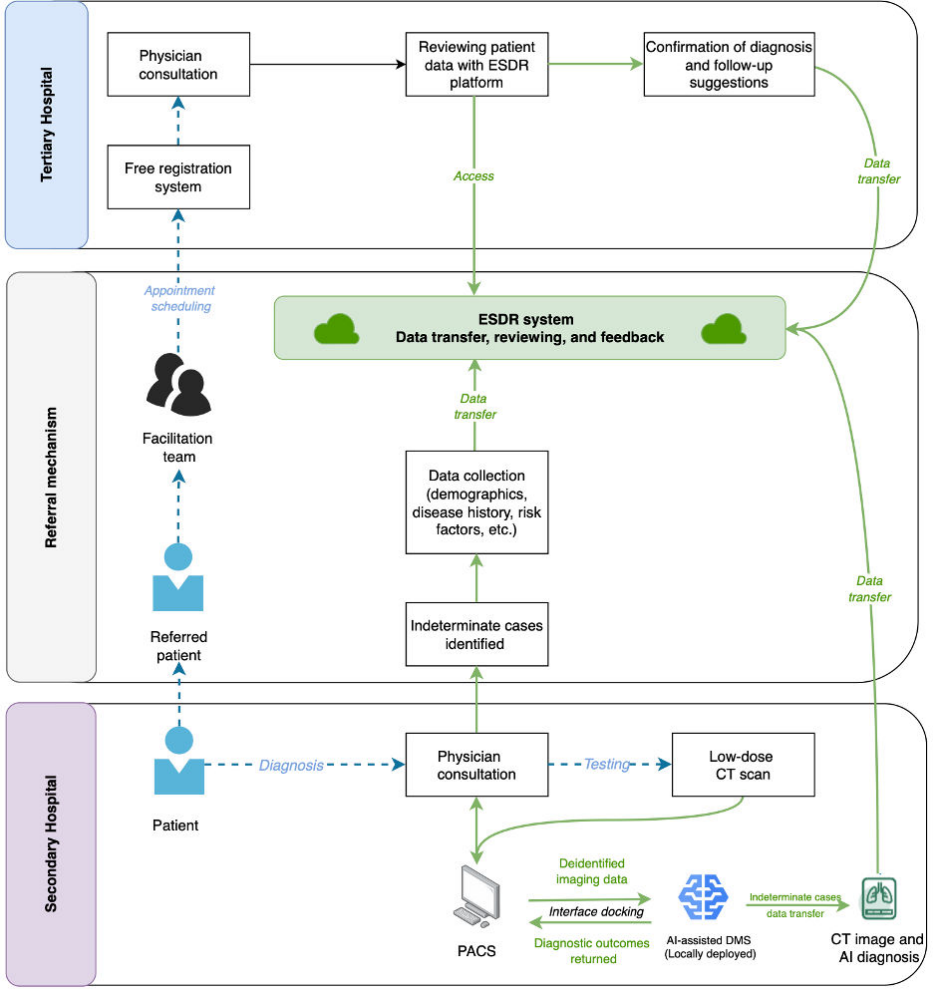
Implementation process flowchart. AI: artificial intelligence; CT: computed tomography; DMS: decision-making software; ESDR: electronic source data repository; PACS: picture archiving and communication system.

The implementation process was illustrated in Figure 1, where the dotted blue line represented patient flow and the solid green line denoted data flow.

The secondary hospital served as the primary site for the implementation of AI-assisted diagnostic software and functioned as the first point of care for patients with suspected pulmonary nodules. Eligible patients presenting with symptoms or undergoing routine cancer screening received an initial clinician consultation, followed by a low-dose CT scan if indicated. The AI-assisted diagnostic software, locally deployed at the secondary hospital, analyzed deidentified imaging data, and the results interfaced directly with the PACS.

When indeterminate cases were identified, physicians at the secondary hospital were responsible for uploading relevant referral information to the ESDR tool, which included:

- Patient demographics: name, date of visit, patient ID, age, ethnicity, sex, phone number, and occupation.
- Clinical symptoms: symptom duration and presentations such as chest pain, cough, or dyspnea.
- Radiological findings: diagnostic conclusions, size, location, morphology, and risk assessment of the primary pulmonary nodule.
- AI diagnostic results and CT images: automatically extracted based on the CT image ID.
- Referral form: documentation of the referral request, reason for referral, and medical insurance information.
- Previous examinations: previous imaging records and other reports, if available.

At the tertiary hospital, a structured referral pathway was established to facilitate the comprehensive evaluation of patients with indeterminate pulmonary nodules. Supported by the ESDR tool, the referral mechanism ensured continuity of care and seamless data flow between institutions. To enhance access to tertiary care, a free registration system was introduced, allowing referred patients to bypass additional registration fees. Moreover, a facilitation team was established to streamline the referral process, assist with appointment scheduling, and provide guidance on accessing tertiary care and navigating the registration system.

Following specialist consultations, the tertiary hospital communicated diagnostic results and follow-up recommendations back to the secondary hospital through the ESDR tool, fostering a continuous feedback loop and building trust in the adoption of AI-assisted diagnostic software.

The integrated strategies were deployed over a 3-month period, from August to November 2024. Following this deployment phase, a transition period was initiated, during which on-site training sessions were held at both hospitals to familiarize physicians with the AI-assisted diagnostic software and the ESDR tool. Concurrently, both systems underwent extensive testing and fine-tuning to ensure effective deployment, with technical challenges addressed and data-sharing protocols refined. Official data collection began in December 2024.

### Study Participants

 The study included both physicians and patients. Eligibility criteria for physicians were as follows:

- Inclusion criteria:
  - Radiologists and respiratory physicians from the secondary hospital, regardless of professional title
  - Respiratory physicians with intermediate or senior professional titles from the tertiary hospital
  - Aged 18 years or older, with no restrictions on sex
  - Possession of a current and legally approved medical license
- Exclusion criteria:
  - Physicians who are unable to provide written informed consent

Patients seeking care or undergoing cancer screening at the secondary hospital were assessed for eligibility for AI-assisted interpretation of CT scans based on the following criteria:

- Inclusion criteria:
  - Age 18 years or older, with no restrictions on sex
  - Underwent a chest CT scan
- Exclusion criteria:
  - History of prior lung surgery

Patients with indeterminate findings were subsequently referred to the tertiary hospital for further evaluation based on specific criteria, including:

- Malignancy risk of the dominant nodule between 5% and 65%
- Dominant pulmonary nodule larger than 8 mm/200 m^3^
- Rapid growth of the dominant nodule size within 6 months
- Family history of lung cancer or personal history of other cancers
- Presence of high-risk factors, including smoking history, age, and occupational or environmental exposure
- Any other conditions for which physicians recommended referral

### Outcomes

As a widely recognized framework for evaluating health care interventions, RE-AIM examines 5 dimensions, including Reach, Effectiveness, Adoption, Implementation, and Maintenance, to support the translation of innovations into routine care [46-48].

1. Reach assesses the engagement, representation, and access of the target population to the intervention.
2. Effectiveness evaluates the intervention’s impact on relevant clinical outcomes.
3. Adoption examines the uptake of the intervention by the intended users or settings.
4. Implementation focuses on the feasibility, usability, and integration of the intervention into existing workflows.
5. Maintenance addresses the long-term sustainability and institutionalization of the intervention.

Given that the current study was designed to assess feasibility and short-term outcomes to inform further refinement of the implementation strategies, the Maintenance dimension was not included. By addressing the remaining dimensions, the RE-AIM framework enabled a multilevel evaluation of the implementation strategies across key stakeholders, including patients, physicians, and health care institutions, providing a comprehensive perspective on their impact. This multidimensional evaluation provided a holistic understanding of the feasibility and effectiveness of the implementation strategies for AI-assisted diagnostic software in pulmonary nodule diagnosis within real-world health care settings, while also identifying opportunities for future optimization and potential scalability.

The specific evaluation indicators are shown in Table 4.

**Table 4.** The RE-AIM outcomes adapted to the evaluation of AI-assisted diagnostic software implementation strategies.

Outcomes	Definitions	Data sources
Reach		
Patient-level		
Representativeness of participants	Differences in patient demographic and clinical characteristics before and after implementation strategy deployment.	Secondary hospital HIS^a^/PACS^b^
AI^c^-assisted screening rate	Proportion of eligible patients who were screened using the AI-assisted diagnostic software.	Secondary hospital PACS/AI software
Referral volume	Total number of patients issued referral notes for tertiary care.	ESDR^d^
Representativeness of referred patients	Demographic and clinical characteristics of patients who were recommended for referral.	Secondary hospital HIS
Provider-level		
Number of physicians who agreed to participate	Number of participating physicians from secondary and tertiary hospitals.	Additional data collection
Effectiveness		
Provider-level		
Change in pulmonary nodules detection rate	Differences in detection rates of pulmonary nodules before and after the deployment of the implementation strategies.	Secondary hospital PACS/AI software
Differences in CT image interpretation and reporting time	Change in average radiologist CT^e^ assessment and reporting time before and after implementation strategy deployment. Interpretation and reporting time = Time of report completion – Time of scan completion	Secondary hospital PACS
Patient-level		
Referral participation rate	Proportion of referred patients who visited the tertiary hospital.	Tertiary hospital HIS
Referral time	Referral time = registration time at the tertiary hospital – referral submission time in the ESDR tool Referral time for patients transferred from the secondary hospital to the tertiary hospital.	Tertiary hospital HIS/ESDR
Adoption		
Provider-level		
AI software adoption rate	Proportion of eligible physicians in the secondary hospital who used AI software.	Questionnaire
ESDR adoption rate	Proportion of eligible physicians in secondary and tertiary hospitals who used the ESDR tool.	Questionnaire
Organizational-level		
Referral acceptance rate	Proportion of patients referred by the secondary hospital who were accepted by the tertiary hospital.	Tertiary hospital HIS
Adoption fit	Degree to which the deployment of the implementation strategies aligns with the strategic priorities of participating hospitals.	Questionnaire
Implementation		
Provider-level		
Ease of use	Physicians’ perceived ease of use of the AI software and ESDR tool in secondary and tertiary hospitals.	Questionnaire
Training coverage	Proportion of physicians trained to use the AI software and ESDR tool in secondary and tertiary hospitals.	Questionnaire
Data transfer completeness	Proportion of complete data transmitted through the ESDR tool.	ESDR
Diagnostic result feedback rate	Proportion of eligible physicians at the tertiary hospital who provided final diagnostic results via the ESDR tool.	ESDR
Diagnostic result review rate	Proportion of eligible physicians at the secondary hospital who reviewed the final diagnostic results of referred patients	Questionnaire
Organizational-level		
Disruptiveness to routine clinical practice	Extent to which the deployment of implementation strategies disrupted routine clinical workflows.	Questionnaire
Referral team success rate	Proportion of referred patients successfully managed by the referral collaboration team during the study period.	Tertiary hospital HIS

a HIS: hospital information system.

b PACS: picture archiving and communication system.

c AI: artificial intelligence.

d ESDR: electronic source data repository.

e CT: computed tomography.

### Data Collection

During the study period, deidentified clinical data were prospectively collected for all patients who met the eligibility criteria for pulmonary nodule screening between December 1, 2024, and March 1, 2025. For patients who met the referral criteria, core referral-related data were collected upon obtaining written informed consent and securely transmitted to the ESDR cloud platform to support subsequent diagnostic evaluation and clinical decision-making.

Implementation outcome data were primarily sourced from the hospital information system (HIS), PACS, AI-assisted diagnostic software, and the ESDR tool. To enhance the comprehensiveness and multidimensionality of the data, key feedback information from participating physicians was also collected through electronic questionnaires (Multimedia Appendix 3).

Historical control data were obtained from the same hospital between December 1, 2023, and March 1, 2024. Key outcome indicators included patient representativeness, differences in pulmonary nodule detection rates, and variations in CT image interpretation and reporting times. These indicators were generally stable over time. Given the consistency within the same health care institutions in terms of diagnostic criteria, diagnostic technologies, and the demographic characteristics, disease staging, and severity of the served populations, the use of a historical control group was deemed comparable.

### Data Analysis

Descriptive statistics for qualitative variables were reported as frequencies and percentages. For quantitative variables, the mean (SD) and median (IQR) were calculated. To compare baseline characteristics between the study participants and historical controls, independent *t* tests were used for continuous variables, while chi-square tests were applied for categorical variables. All statistical tests were 2-sided, and statistical significance was set at *P* less than .05.

## Results

### Reach

At the provider level, physicians from relevant clinical departments at both the secondary and tertiary hospitals were invited to participate in the study. Specifically, 6 radiologists and 9 respiratory physicians from the secondary hospital, along with 13 respiratory physicians from the tertiary hospital, were approached. Of those invited, all 15 physicians from the secondary hospital and 8 respiratory physicians from the tertiary hospital agreed to participate. The demographic and professional characteristics of the participating physicians from both institutions are summarized in Table 5.

**Table 5.** Demographic and professional characteristics of participating physicians.

Characteristic	Secondary hospital (n=15)	Tertiary hospital (n=8)
Sex, n (%)		
Male	7 (46.7)	5 (62.5)
Female	8 (53.3)	3 (37.5)
Age (y), median (IQR)	44 (38.5‐49.5)	44.5 (36.5‐50.5)
Highest level of educational attainment, n (%)		
Bachelor’s degree	12 (80)	0 (0)
Master’s degree	3 (20)	4 (50)
Doctoral degree	0 (0)	4 (50)
Professional title, n (%)		
Junior	1 (6.7)	—^a^
Intermediate	6 (40)	4 (50)
Associate senior and above	8 (53.3)	4 (50)

a Not applicable.

At the patient level, a total of 1375 patients underwent chest CT examinations at the secondary hospital during the study period. The mean age of patients was 54 (SD 16.6) years, and 604 (43.9%) were male. The mean maximum diameter of the dominant pulmonary nodules detected was 5.4 (SD 2.3) mm, with 953 (69.3%) classified as solid nodules. Compared with the historical control group, there were no statistically significant differences in patient demographics or clinical characteristics of pulmonary nodules (Table 6).

Of the patients who underwent chest CT examinations, 84 (6.1%) were excluded based on the predefined exclusion criteria, including 76 patients under 18 years of age and 8 patients with a history of prior lung surgery. Among the 1291 patients who met the inclusion criteria, 85.6% of chest CT images were successfully analyzed using the AI-assisted pulmonary nodule diagnostic software.

During the study period, 33 patients met the predefined referral criteria. Of these, 25 (75.8%) patients provided written informed consent and were referred to the tertiary hospital. The median age of the referred patients was 63 (IQR 32‐66) years, and 44% (11/25) were male. The median maximum diameter of the dominant pulmonary nodules was 9 (IQR 8‐11) mm, with 28% (7/25) identified as solid nodules. Additionally, 32% (8/25) of referred patients had a history of other pulmonary diseases, 8% (2/25) had a history of cancer, and 24% (6/25) reported a history of smoking (Table 7).

**Table 6.** Comparison of demographic and clinical characteristics of pulmonary nodules.

Characteristic	Intervention group (n=1375)	Historical control group (n=1591)
Age (y), mean (SD)	54 (16.6)	53.2 (19.5)
Sex, n (%)		
Male	604 (43.9)	743 (46.7)
Female	771 (56.1)	848 (53.3)
Size (mm), mean (SD)	5.4 (2.3)	5.1 (2)
Density, n (%)		
Solid	953 (69.3)	1151 (72.3)
Subsolid^a^	422 (30.7)	440 (27.7)

a Including part-solid nodules and pure ground-glass nodules.

**Table 7.** Demographic and clinical characteristics of patients who consented to referral.

Characteristic	Patients who consented to referral (n=25)
Age (y), median (IQR)	63 (32‐66)
Sex, n (%)	
Male	11 (44)
Female	14 (56)
Other pulmonary diseases, n (%)	
Yes	8 (32)
Chronic obstructive pulmonary disease	1 (12.5)
Pneumonia	5 (62.5)
Asthma	2 (25)
No	17 (68)
Cancer history, n (%)	
Yes	2 (8)
No	23 (92)
Smoking history, n (%)	
Yes	6 (24)
No	19 (76)
Size of dominant nodule (mm), median (IQR)	9 (8-11)
Density of dominant nodule, n (%)	
Solid	7 (28)
Subsolid^a^	18 (72)

a Including part-solid nodules and pure ground-glass nodules.

### Effectiveness

At the provider level, a total of 896 patients at the secondary hospital were identified with at least 1 pulmonary nodule, including micronodules, small nodules, intermediate-sized nodules, and mass-like lesions, resulting in an overall detection rate of 65.2%. The mean time for radiologists to assess and report chest CT images was 24.1 (SD 13.5) minutes. In comparison, during the historical control period, the overall pulmonary nodule detection rate was 32.4% (*P*<.001), and the mean CT image assessment and reporting time was significantly longer (30.4, SD 19.7 min; *P*<.001).

At the patient level, of the 25 individuals who consented to referral, 22 successfully completed the referral process by visiting the tertiary hospital, yielding a referral completion rate of 88%. The median interval from referral submission to hospital visit was 3 (IQR 2‐4) days. The 3 patients who did not complete the referral included 2 males and 1 female, with a median age of 67 years. The median maximum diameter of their dominant pulmonary nodules was 13 mm, and 1 had a documented history of cancer.

### Adoption

At the secondary hospital, an electronic questionnaire (Multimedia Appendix 3) was distributed via email and QR code to 9 respiratory physicians and 6 radiologists who participated in the study. All 15 physicians completed the questionnaire, yielding a response rate of 100%. According to the survey, 7/9 (77.8%) of respiratory physicians and all radiologists had used the AI-assisted pulmonary nodule diagnostic software, while 5/9 (55.6%) of respiratory physicians reported using the ESDR tool. In terms of perceived alignment with institutional goals, all respondents agreed that the AI-assisted diagnostic software supported the hospital’s core objectives, and 6/9 (66.7%) of respiratory physicians believed that the use of the ESDR tool was consistent with those objectives.

At the tertiary hospital, all 22 referred patients who arrived were successfully received, resulting in a referral acceptance rate of 100%. Among the 8 participating respiratory physicians, the electronic questionnaire also achieved a 100% response rate. Of these respondents, 5/8 (62.5%) reported prior use of the ESDR tool, and 7/8 (87.5%) indicated that its use aligned with the hospital’s strategic priorities.

### Implementation

In terms of usability, 11/13 (84.6%) of radiologists and respiratory physicians at the secondary hospital who had used the AI-assisted diagnostic software reported that it was easy to use. In contrast, among respiratory physicians who had experience with the ESDR tool, only 3/5 (60%) at the secondary hospital and 4/5 (80%) at the tertiary hospital perceived the tool as user-friendly.

Although both the AI-assisted diagnostic software and the ESDR tool achieved 100% training coverage across the two hospitals, the completeness of referral data collected via the ESDR tool remained suboptimal. Only 28% (7/25) of referred cases had complete data entries, with comprehensive datasets available for 7 patients. Missing information was primarily concentrated in key fields, including clinical symptoms, diagnostic conclusion, and referral-related variables such as insurance information. Despite a relatively high CT image transmission success rate of 90.9% (20/22), system-level failures persisted. Among the 22 patients who successfully completed the referral process, two experienced data transmission failures due to PACS system malfunctions at the secondary hospital, which prevented the upload of AI-generated results and CT images to the ESDR platform. Consequently, these patients were required to carry physical CT films to the tertiary hospital for further diagnostic evaluation.

With respect to integration into clinical workflows, 93.3% (14/15) of physicians at the secondary hospital reported that the AI-assisted diagnostic software did not interfere with routine clinical practice. However, 55.6% (5/9) of respiratory physicians indicated that the ESDR tool imposed a substantial burden on daily tasks. The primary challenge was the lack of interoperability between the ESDR system and the hospital’s internal information systems, which required physicians to manually access the platform via an external web link to upload or retrieve patient data. This additional step increased workload and disrupted workflow efficiency. As a result, continuity of care was affected. Among the 9 respiratory physicians at the secondary hospital, only 44.4% (4/9) reviewed the final diagnostic results of referred patients via the ESDR tool.

At the tertiary hospital, the referral coordination team successfully facilitated the referral process for all 22 patients, achieving a referral completion rate of 100%. Nevertheless, only 50% (4/8) of respiratory physicians uploaded final diagnostic results through the ESDR system. Moreover, 62.5% (5/8) reported workflow disruptions due to challenges similar to those in the secondary hospital, particularly the reliance on external network access, which increased complexity and time demands.

## Discussion

### Principal Findings 

This study designed, implemented, and evaluated an integrated strategy specifically tailored to support the effective implementation of AI-assisted diagnostic software within the context of China’s HMS. In contrast to prior studies that primarily focused on identifying implementation barriers [49-53], this study developed a context-specific implementation framework and demonstrated tangible improvements in both diagnostic capacity and referral processes at the secondary care level. During the study period, 85.6% of chest CT images at the secondary hospital were analyzed using AI-assisted diagnostic software. As a result, the overall detection rate of pulmonary nodules significantly increased from 32.4% in the historical control group to 65.2% in the intervention group (*P*<.001), indicating a significant enhancement in diagnostic capacity. In addition, the introduction of a standardized referral pathway supported high levels of referral compliance. Supported by a dedicated coordination team, 88% of the 25 patients identified as referral candidates successfully completed the referral process and received timely diagnostic and therapeutic services at the tertiary hospital. The median referral time was approximately 3 days, indicating efficient coordination and strong adherence to the referral process. Moreover, 90.9% of CT images acquired at the secondary hospital were successfully transmitted to the tertiary hospital via the ESDR platform, enabling receiving physicians to access imaging data in advance and facilitating more accurate and timely diagnoses. The high transmission success rate and streamlined interhospital data exchange strengthened institutional coordination while enhancing the continuity of diagnostic and treatment services. Study outcomes collectively supported the feasibility of scaling AI-assisted diagnostic software technologies across lower-tier health care settings and demonstrated their value in strengthening referral processes and optimizing care pathways within the hierarchical health care system.

 The findings aligned with a growing global consensus on the strategic importance of deploying AI in lower-tier health care settings. Internationally, the implementation of AI-assisted diagnostic software technologies varies widely, influenced by differences in software types, national health care infrastructure, policy environments, and the maturity of health information systems. Although technical and organizational challenges remain, recent research has increasingly emphasized the potential of AI to improve diagnostic accuracy for common diseases, streamline clinical workflows, and optimize health care resource use, particularly in settings such as community health centers and secondary hospitals [38,54-57]. AI technologies are especially valuable in supporting primary care providers by enabling large-scale patient management, improving early disease detection, and reducing diagnostic errors in environments where clinical experience and infrastructure are limited [58-60].

Despite the growing recognition of these benefits, most existing studies have concentrated on evaluating clinical performance metrics such as sensitivity, specificity, and referral accuracy [61-63] or have identified implementation challenges like inadequate referral adherence [63]. Relatively few studies have developed and empirically validated comprehensive implementation strategies adapted to lower-tier clinical environments. This gap highlights the need to move beyond performance-oriented evaluations toward theory-informed investigations of how AI technologies can be effectively integrated into routine care.

 From an implementation science perspective, this study extended the application of ERIC strategies by demonstrating how their mechanisms of action vary across health system contexts. While ERIC strategies have largely been derived from Western health systems where hospitals and clinical units have substantial authority to determine implementation priorities, allocate resources, and adapt technologies to local workflows, recent evidence indicates that these strategies are increasingly applied in low- and middle-income country contexts, often requiring contextual adaptation. A systematic review of ERIC strategy use in low- and middle-income countries found that the majority of the 73 ERIC strategies have been selectively adapted to address diverse implementation barriers within resource-limited health systems, underscoring the importance of context-specific specification and tailoring to address structural and policy barriers distinct from those in high-income settings [64]. Consistent with this theoretical framing, the barriers identified in the present study were shaped primarily by system-level regulatory and structural constraints within China’s HMS, leading to prioritization of system-level and inter-organizational ERIC strategies over those commonly emphasized in Western contexts. By explicitly linking CFIR-identified determinants to contextually adapted ERIC strategies, this study underscores the importance of selecting implementation strategies based on the level at which barriers operate. This theory-informed, context-sensitive approach informed the study’s emphasis on feasibility and implementation processes rather than clinical performance outcomes alone.

The need for such implementation-focused research is particularly urgent in China, where structural imbalances within the HMS continue to place substantial pressure on tertiary hospitals. According to the Statistical Bulletin on the Development of China’s Health Sector released by the National Health Commission, outpatient and emergency visits to tertiary hospitals rose from 2.06 billion in 2019 to 2.63 billion in 2023, a 27.7% increase over four years, indicating a sustained upward trend [65,66]. In contrast, patient volume at primary health care institutions has remained relatively stable [67], underscoring the continued underutilization of lower-tier facilities and a growing imbalance in resource allocation. To address these structural inefficiencies, the implementation strategies proposed in this study focused on enhancing diagnostic capacity at the secondary care level and reducing unnecessary reliance on tertiary hospitals.

 Furthermore, inadequate referral mechanisms remain a major challenge in China’s HMS [10,68,69]. A cost-effectiveness analysis of AI-assisted screening for diabetic retinopathy in community health centers in China underscored the pivotal role of referral adherence in determining the overall value of AI-based telemedicine screening [70]. The results indicated that, in the absence of improved follow-up among patients with suspected disease, the adoption of AI tools might offer no significant advantage over manual grading in telemedicine screening, as reduced long-term effectiveness and health utility would offset potential cost savings. The persistence of inadequate referrals partially reflects systemic challenges such as fragmented health information and the lack of standardized referral protocols [71-73]. These systemic gaps could compromise continuity of care, delay timely diagnosis and treatment, and reduce the overall efficiency of health care delivery [74,75]. In recent years, the application of digital tools to referral processes has shown promise in addressing these challenges by facilitating data integration and strengthening communication between institutions [76,77]. Building on this evidence, the current study integrated the ESDR platform into the referral process, which facilitated more efficient data exchange, reduced delays, and enhanced the scalability of referral pathways in lower-tier health care settings. Additionally, the patient-centered design of the referral process helped reduce administrative, logistical, and financial burdens associated with repeated patient registration and diagnostic procedures, promoting timely and high-quality care.

Taken together, the integrated implementation strategies developed in this study reinforced the role of AI in supporting stratified care within China’s HMS. Within this framework, community and secondary hospitals are empowered to perform initial AI-assisted screenings to identify suspected or high-risk cases, which are subsequently referred in a timely and structured approach to collaborating tertiary hospitals for further evaluation and treatment. This allows tertiary hospitals to focus their resources on complex and critical cases, while lower-tier institutions effectively manage less severe conditions. The resulting model of decentralized screening and streamlined referral enhances patient flow across care levels, improves the responsiveness of service delivery, and ensures the timely transition of patients to appropriate levels of care. By enhancing clinical workflow efficiency and improving the use of health care resources, this approach contributes to a more functional HMS and facilitates the delivery of care that is well-coordinated, timely, and equitable across different levels of the health care system.

 However, several barriers were identified that might hinder the broader effectiveness and scalability of the implementation strategies. Notably, 14.4% of cases were not analyzed by the AI software, a gap that appeared to be primarily attributable to operational and workflow-related factors rather than algorithmic limitations. These factors may include interruptions in data transmission, timing mismatches between image acquisition and AI processing, and clinician-driven decisions not to activate the AI tool in specific clinical scenarios. In addition, data incompleteness emerged as a major challenge, as only 28% of referral records contained all required information. Missing data were especially prevalent in key fields, including clinical symptoms, diagnostic conclusions, and insurance information. Poorly structured or incomplete referral documentation has been reported as a main barrier to effective communication and care coordination across different tiers of health care institutions in China [77]. These gaps would impede the clinical capacity of physicians at the tertiary hospital to perform comprehensive clinical assessments, potentially affecting diagnostic accuracy. This finding was consistent with previous studies that have emphasized the ongoing fragmentation of HISs in China, frequently functioning in isolation due to the absence of standardized data standards and limited interoperability across institutions [78,79]. Further, disruptions to routine clinical workflows were commonly reported as a main barrier to the effective adoption of digital health innovations [80-82]. Due to administrative constraints, data security concerns, and technical limitations, the ESDR platform could not be integrated with existing hospital systems (eg, HIS and PACS) and was deployed on a public cloud. As a result, clinicians were required to manually navigate multiple platforms, increasing operational complexity and workload. To address these challenges, future efforts should focus on enhancing the interoperability and operational efficiency of interhospital data exchange systems through seamless integration [78,83,84]. Such interoperability would reduce the operational burden associated with navigating multiple systems and manual data entry, while enhancing the accuracy, completeness, and timeliness of data transmission. Additionally, there is an urgent need for continued examination to determine the adaptability, effectiveness, and long-term sustainability of implementation strategies across diverse health care settings [85]. Such evidence is essential for supporting the widespread, equitable, and context-specific integration of AI technologies into routine clinical practice and for fully realizing their potential to improve diagnostic accuracy, streamline care coordination, and enhance overall health system performance. 

### Limitations

This study had several limitations that should be addressed in future research. The implementation was conducted in a single urban setting in Beijing, involving one secondary and one tertiary rehabilitation hospital, and focused exclusively on pulmonary nodule screening. Although the findings offered clinically relevant insights into the feasibility and potential refinement of implementation strategies, the complexity of other health care settings, such as community health centers or rural clinics, and the applicability to different disease domains remains insufficiently addressed.

In addition, this study did not include a formal organizational-level cost analysis. Although conducted in a real-world clinical environment, the implementation represented an early-phase deployment in a resource-constrained secondary hospital, supported through research collaboration and vendor participation. Under these transitional conditions, short-term cost estimates would be unlikely to reflect routine, institutionally financed adoption. Moreover, key cost drivers, including long-term maintenance and system integration costs, changes in personnel workload, and downstream effects on referral patterns and health care utilization, require longer follow-up periods and more stable operational contexts to be meaningfully assessed.

Further, the single-center, pre-post study design limited the ability to fully account for temporal confounding and restricted external validity. Although the preimplementation and postimplementation periods were matched by calendar months to minimize seasonal variation, unmeasured temporal factors, such as changes in patient characteristics, institutional practices, or broader system-level influences, may still have affected the observed outcomes. Moreover, as the current study primarily focused on feasibility and areas for implementation optimization, both short-term and long-term sustainability were not evaluated. To improve generalizability and strengthen internal validity, future research should adopt multicenter, prospective study designs incorporating concurrent control groups and consider Type II hybrid implementation-effectiveness approaches to enable simultaneous evaluation of implementation outcomes, clinical impact, cost-effectiveness, and long-term scalability of AI-assisted diagnostic software.

### Conclusion

This study developed and evaluated an integrated strategy to support the effective implementation of AI-assisted diagnostic software for pulmonary nodule screening in a secondary hospital in China. The findings demonstrated enhanced screening capacity, referral efficiency, and interhospital coordination, highlighting the potential of AI to strengthen the hierarchical health care delivery model. As an early-phase implementation study, these results should be interpreted as preliminary and should primarily be used to inform and refine the proposed implementation strategies. To support future scalability and real-world impact, targeted actions are needed, including promoting standardized interfaces between HISs and third-party platforms to improve interoperability, strengthening governance mechanisms for secure data sharing, and optimizing referral workflows across care levels. Together, these measures offer a practical pathway toward a more precise, efficient, and sustainable health care system, while contributing to more equitable allocation of medical resources. Future research should prioritize multicenter studies and long-term evaluations to inform the broader adoption of AI technologies across diverse clinical settings.

## Supplementary material

10.2196/76002 Multimedia Appendix 1 Consolidated Framework for Implementation Research-Expert Recommendations for Implementing Change mapping process and results.

10.2196/76002 Multimedia Appendix 2 Functionalities of the artificial intelligence–assisted diagnostic software.

10.2196/76002 Multimedia Appendix 3 Physician questionnaire.

## Abbreviations

AI: artificial intelligence
CFIR: Consolidated Framework for Implementation Research
CT: computed tomography
ERIC: Expert Recommendations for Implementing Change
ESDR: electronic source data repository
HIS: hospital information system
HMS: hierarchical medical system
PACS: picture archiving and communication system
