# Supplementary material for: Integrated Implementation Strategies to Promote the Use of AI-Assisted Diagnostic Software for Lung Nodule Screening in China: Process Evaluation Based on the RE-AIM Framework
Source: JMIR Form Res. 2026 Mar 24;10:e76002. doi: 10.2196/76002 (PMC13011999; doi:10.2196/76002)
Supplement: Multimedia Appendix 1 [file formative-v10-e76002-s001.docx]

**Multimedia Appendix 1 CFIR-ERIC mapping process and results**

Using the CFIR-ERIC mapping tool, a systematic selection of ERIC implementation strategies was conducted to address five key implementation barriers previously identified. These barriers included: (1) relative advantage (unsatisfactory clinical performance at tertiary hospitals); (2) goals and feedback (misalignment between software functions and goals of tertiary hospitals); (3) reflecting and evaluating (lack of performance feedback and evaluation) (4) external collaboration (lack of a collaborative network between primary or secondary and tertiary hospitals); and (5) external policies and incentives (lack of information security measures and certification).

The matching results are presented in the form of both individual and cumulative percentages to reflect the degree of endorsement for each ERIC implementation strategy in addressing specific CFIR-identified barriers. The individual percentage represents the suitability of each implementation strategy in relation to a single CFIR barrier, while the cumulative percentage captures the overall appropriateness and prioritization of each ERIC strategy when multiple barriers interact.

Based on the level of endorsement, the ERIC strategies are categorized into three tiers. Tier 1 (marked in green): endorsement ≥ 50%, indicating that the strategy is considered highly feasible and adaptable across most implementation contexts. Tier 2 (marked in yellow): endorsement between 20% and 49.9%, suggesting that the strategy may be feasible in specific contexts but likely requires further environmental adaptation and refinement. Tier 3 (marked in white): endorsement < 20%, indicating low feasibility in the given barrier context and typically not prioritized unless supported by additional validation or context-specific evidence.

Supplemental Table 1 presented the implementation strategies identified by the CFIR-ERIC mapping tool with cumulative percentages exceeding 50%.

| ERIC Strategies | Cumulative  Percent | Relative advantage | Cosmopolitanism | External Policy & Incentives | Goals & Feedback | Reflecting & Evaluating |
| --- | --- | --- | --- | --- | --- | --- |
| Build a coalition | **132%** | 14% | 62% | 33% | 15% | 8% |
| Audit and provide feedback | **127%** | 10% | 0% | 0% | 61% | 56% |
| Develop and implement tools for quality monitoring | **105%** | 7% | 0% | 11% | 27% | 60% |
| Identify and prepare champions | **103%** | 45% | 15% | 22% | 12% | 8% |
| Capture and share local knowledge | **102%** | 17% | 23% | 26% | 12% | 24% |
| Inform local opinion leaders | **91%** | 28% | 15% | 22% | 18% | 8% |
| Conduct local consensus discussions | **88%** | 24% | 15% | 22% | 18% | 8% |
| Facilitate relay of clinical data to providers | **86%** | 10% | 0% | 4% | 36% | 36% |
| Alter incentive/allowance structures | **83%** | 28% | 0% | 41% | 15% | 0% |
| Develop and organize quality monitoring systems | **83%** | 3% | 0% | 15% | 24% | 40% |
| Organize clinician implementation team meetings | **78%** | 14% | 0% | 0% | 36% | 28% |
| Develop a formal implementation blueprint | **77%** | 7% | 4% | 7% | 42% | 16% |
| Conduct educational meetings | **76%** | 24% | 12% | 15% | 21% | 4% |
| Involve executive boards | **75%** | 3% | 23% | 41% | 0% | 8% |
| Create a learning collaborative | **73%** | 7% | 31% | 15% | 12% | 8% |
| Use advisory boards and workgroups | **72%** | 10% | 35% | 15% | 12% | 0% |
| Promote network weaving | **71%** | 3% | 50% | 11% | 6% | 0% |
| Develop academic partnerships | **68%** | 0% | 50% | 11% | 3% | 4% |
| Visit other sites | **67%** | 21% | 38% | 7% | 0% | 0% |
| Facilitation | **64%** | 10% | 12% | 4% | 18% | 20% |
| Conduct local needs assessment | **63%** | 34% | 12% | 7% | 6% | 4% |
| Purposely reexamine the implementation | **62%** | 7% | 4% | 11% | 12% | 28% |
| Assess for readiness and identify barriers and facilitators | **61%** | 24% | 15% | 4% | 6% | 12% |
| Obtain formal commitments | **53%** | 7% | 19% | 15% | 12% | 0% |

Supplemental Table 1 CFIR-ERIC mapping results
