# Supplementary material for: Integrated Implementation Strategies to Promote the Use of AI-Assisted Diagnostic Software for Lung Nodule Screening in China: Process Evaluation Based on the RE-AIM Framework
Source: JMIR Form Res. 2026 Mar 24;10:e76002. doi: 10.2196/76002 (PMC13011999; doi:10.2196/76002)
Supplement: Multimedia Appendix 2 [file formative-v10-e76002-s002.docx]

Multimedia Appendix 2 Functionalities of the AI-assisted diagnostic software

The AI-assisted screening system for pulmonary nodules (HY Medical Technology Co., Ltd, Beijing, China) was approved by the National Medical Product Administration in 2022 and obtained CE certification in 2020. A proprietary 3D convolutional neural network (CNN) for object detection, utilizing random initialization, supervised training, and model ensemble techniques, was employed to accurately detect pulmonary nodules. The underlying CNN algorithm was trained on a dataset of 4638 chest CT scans from five hospitals across two provinces in China, including patients over 18 years old with a balanced sex distribution (Supplemental Fig. 2). The CT imaging equipment used in the dataset was primarily from Siemens, GE, and Philips, reflecting typical clinical usage. All scans had a slice thickness below 2.5 mm, with most between 1 mm and 2 mm.


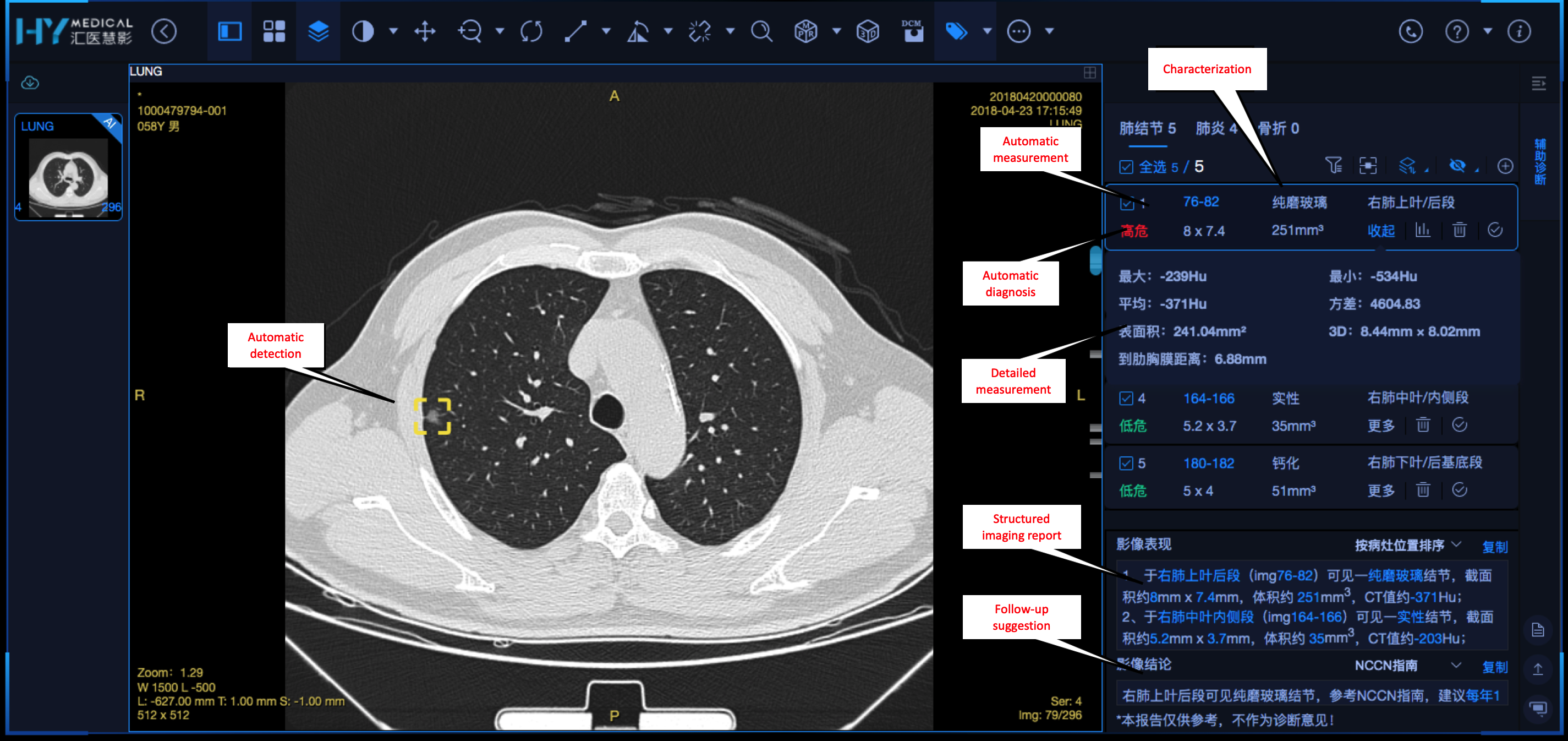


**Supplemental Fig. 1 Functions of the AI-assisted screening system for pulmonary nodules**

- **Annotations:**
  - Automatic detection: Scans thin-slice lung CT images to locate and mark pulmonary nodules automatically and rapidly, providing a one-click guide function.
  - Automatic measurement: Accurately segments nodule contours, automatically measuring lesion diameter, volume, density, surface area, and histogram information.
  - Automatic characterization: Automatically classifies the nature of the nodule, including solid, part-solid, ground-glass, and calcified nodules.
  - Automatic diagnosis: Predicts the risk level of the nodule (high, medium, low) based on international guidelines and provides the probability of malignancy.
  - Automatic navigation: Supports MPR (Multi-Planar Reconstruction) display for simultaneous sagittal, coronal, and axial views.
  - One-Click report generation: Generates a structured textual description and imaging conclusions with a single click.
  - Professional follow-Up suggestion: Provides follow-up suggestions according to international guidelines.


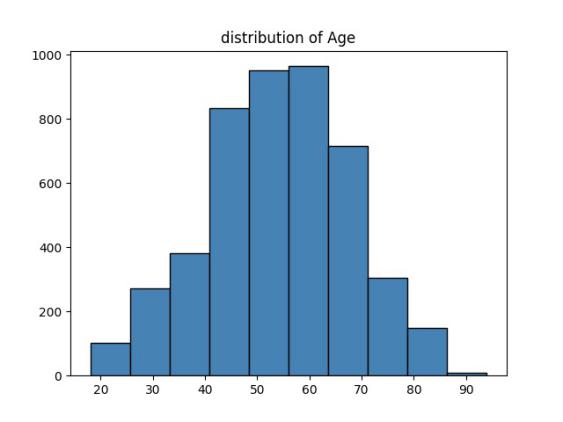

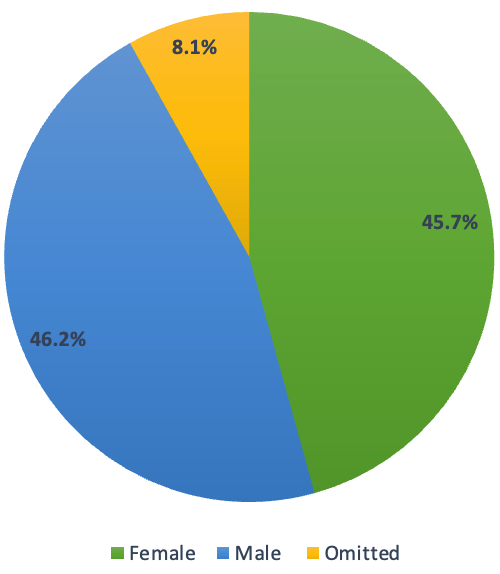


(a) Distribution of patient age (b) Distribution of patient sex


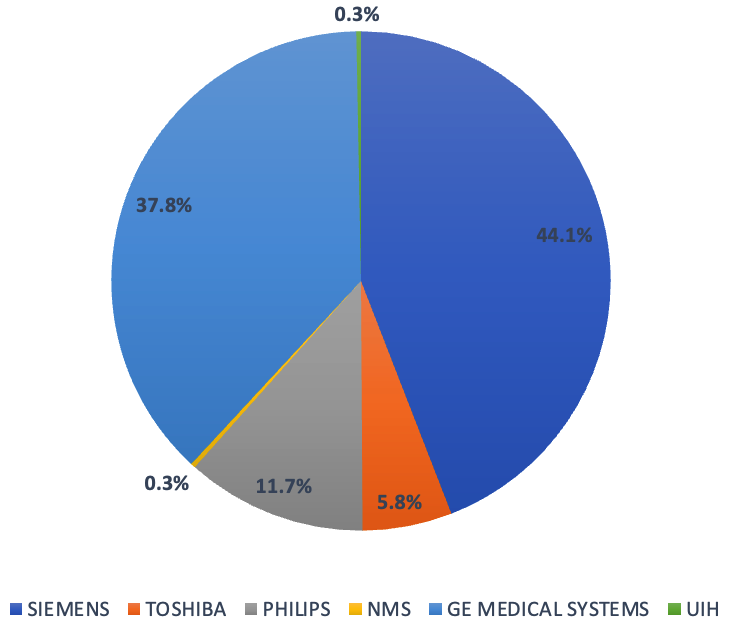

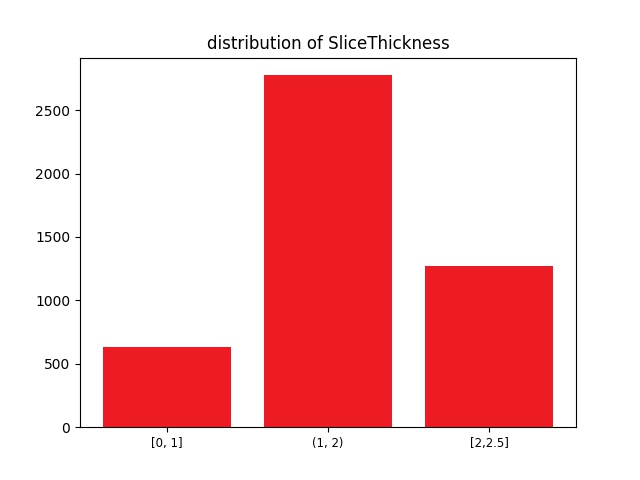


(c) Distribution of CT manufacturer (d) Distribution of CT scan slice thickness

**Supplemental Fig. 2 Characteristics of the training dataset for the** **AI-assisted screening system for pulmonary nodules**
