# Supplementary material for: Integrated Implementation Strategies to Promote the Use of AI-Assisted Diagnostic Software for Lung Nodule Screening in China: Process Evaluation Based on the RE-AIM Framework
Source: JMIR Form Res. 2026 Mar 24;10:e76002. doi: 10.2196/76002 (PMC13011999; doi:10.2196/76002)
Supplement: Multimedia Appendix 3 [file formative-v10-e76002-s003.docx]

Multimedia Appendix 3 Physician questionnaire

**Integrated implementation strategies to promote** **the use of artificial intelligence-assisted diagnostic software for lung nodule screening in China – a process evaluation based on the RE-AIM framework: physician Survey**

**Introduction**

Dear healthcare providers,

In recent years, national policy guidelines in China have increasingly advocated for the integration of real-world clinical data into healthcare practice and the adoption of artificial intelligence (AI) to drive medical innovation. To support the development of a practical and effective framework for implementing AI-assisted diagnostic software, particularly aimed at improving diagnostic capacity in secondary hospitals and advance the hierarchical medical system, the China Rehabilitation Research Center is conducting a process evaluation guided by the RE-AIM framework. The study focused on implementation strategies for AI-assisted diagnostic software, using uncertain pulmonary nodules as a representative case.

We invite you to complete this survey to assess the effectiveness of the implementation strategies. The questionnaire is anonymous, and all responses will be kept strictly confidential. Participation is entirely voluntary, and you may withdraw at any time without any negative consequences. Your responses are crucial in supporting the application of AI-assisted diagnostic software in China’s clinical environment. The estimated time to complete the survey is approximately 5 minutes.

For any inquiries regarding this study, please contact:

Email: ceceliao94@outlook.com

Ethics Office, China Rehabilitation Research Center: +86-10-87020512

Please indicate whether you agree to participate in this survey:

① I agree (Proceed to Part I: Basic Information)

② I do not agree (Proceed to Part V: Closing Statement)

**Part I: Basic Information**

1.1 What is your gender?

① Male ② Female

1.2 What is your age? _____ years

1.3 What is the highest level of academic degree you have attained?

① Associate/Bachelor’s Degree

② Master’s Degree

③ Doctoral Degree

1.4 What is your professional title?

① Junior ② Intermediate ③ Senior or above

1.5 Which medical institution are you affiliated with?

① Fengtai Rehabilitation Hospital of Beijing → (Proceed to 1.6)

② China Rehabilitation Research Center → (Proceed to Part IV)

1.6 Which department do you work in?

① Radiology → (Proceed to Part II)

② Respiratory Medicine → (Proceed to Part III)

**Part II: Evaluation of the Implementation of the AI-Assisted lung Nodule Diagnostic Software**

2.1 Have you received training on the use of the AI-assisted lung nodule diagnostic software?

① Yes ② No

2.2 Have you used the AI-assisted software for diagnosis?

① Yes → (Proceed to 2.2.1)

② No → (Proceed to 2.3)

2.2.1 Do you consider the software easy to use?

① Yes ② No

2.2.2 Do you feel the software interferes with your routine clinical work?

① Yes (Please specify the reason) ② No

2.3 Do you believe that the deployment of the software aligns with your hospital’s core objectives (e.g., early detection, diagnosis, treatment, and rehabilitation of lung cancer)?

① Yes ② No

**End of this section. Thank you for your time.**

**Part III: Evaluation of the Implementation of the AI Software and ESDR Tool**

**3.1 AI Software: Same questions as in Part II**

**3.2 ESDR Tool:**

3.2.1 Have you received training on the use of the ESDR tool?

① Yes ② No

3.2.2 Have you used the ESDR tool for inter-hospital data transfer?

① Yes → (Proceed to 3.2.2.1)

② No → (Proceed to 3.3)

3.2.2.1 Do you find the ESDR tool easy to use?

① Yes ② No

3.2.2.2 Do you think using the ESDR tool interferes with your clinical work?

① Yes (Please specify the reason) ② No

3.3 Do you believe the deployment of the ESDR tool aligns with your hospital’s core objectives (e.g., supporting early detection and coordinated care for lung cancer)?

① Yes ② No

3.4 Have you used the ESDR tool to review the final diagnostic results of referred patients?

① Yes ② No

**End of this section. Thank you for your time.**

**Part IV: Evaluation of the Referral Mechanism and ESDR Tool**

4.1 Have you received training on the ESDR tool?

① Yes ② No

4.2 Have you used the ESDR tool to review referral patient data?

① Yes → (Proceed to 4.2.1)

② No → (Proceed to 4.3)

4.2.1 Do you find the ESDR tool easy to use?

① Yes ② No

4.2.2 Do you think using the ESDR tool interferes with your clinical work?

① Yes (Please specify the reason) ② No

4.3 Do you believe the deployment of the ESDR tool aligns with your hospital’s core objectives (e.g., facilitating the diagnosis of complex lung nodules and reducing redundant testing)?

① Yes ② No

4.4 Have you used the ESDR tool to upload final diagnostic results for referred patients?

① Yes ② No

**Part V: Closing Statement**

**This concludes the survey. We sincerely thank you for your valuable time and contribution to this study.**
